# Neural time course of processing socially meaningful symbolic cues: ERP differences between national-symbol and neutral-object icons

**DOI:** 10.3389/fnhum.2026.1838401

**Published:** 2026-06-22

**Authors:** Zhiqiang Li

**Affiliations:** 1 Center for Ideological and Political Education, Northeast Normal University, Changchun, China; 2 Business & Tourism Institute, Hangzhou Polytechnic University, Hangzhou, China

**Keywords:** event-related potentials, N400-range negativity, national symbols, social cognition, symbolic cues, visual perception

## Abstract

Socially meaningful symbolic cues are common in everyday visual environments, yet the timing with which they diverge from neutral objects during neural processing remains unclear. This study examined the temporal dynamics of one culturally meaningful class of symbolic cues, national-symbol icons, using event-related potentials (ERPs). Thirty-four Chinese university students completed a within-subject ERP task in which 30 national-symbol icons and 30 neutral-object icons were each presented twice. Participants indicated whether they would be willing to share each icon and then rated pleasantness, arousal, and patriotic feeling. Mean ERP amplitudes were analyzed in three predefined time windows: N1 (100–150 ms), N400-range negativity (250–400 ms), and late positive potential (LPP; 550–750 ms). National-symbol icons were associated with greater willingness to share and higher ratings of pleasantness, arousal, and patriotic feeling than neutral-object icons. At the neural level, national-symbol icons elicited a more negative N1 and a larger N400-range negativity. The LPP difference was not statistically significant, although national-symbol icons showed numerically greater positivity. Overall, national-symbol icons differed from neutral-object icons in more than one predefined ERP time window, with the clearest effect in the 250–400 ms interval. The findings are broadly consistent with enhanced mid-latency processing of socially shared meaning in one culturally meaningful class of symbolic cues, although this interpretation remains tentative because the present study provides condition-level behavioral convergence rather than definitive participant-level brain–behavior validation.

## 1 Introduction

Flags, national emblems, commemorative architecture, and other recognizable nation-related icons are more than political or cultural markers. In everyday life, they can function as socially meaningful symbolic cues that condense collective belonging, culturally shared values, and broader social meaning. Classic work on nation-states and collective symbols has long suggested that such stimuli help organize group attachment and public meaning (Smith, 1991; Kolstø, 2006; Butz, 2009). More recent work on social perception and visible appearance cues likewise indicates that symbolic markers can shape how people interpret individuals, groups, and social identities (Carlston and Mae, 2007; Hester and Hehman, 2023; Camacho and Abouras, 2025). National symbols therefore provide a useful, but still understudied, stimulus class for examining how socially meaningful visual cues unfold over time in the human brain.

Behavioral research suggests that national and other identity-relevant cues can influence judgment, evaluation, and behavior. Exposure to national flags, for example, can bias political thought and action (Hassin et al., 2007), and identity-linked cues have been connected to person perception, cooperation, and group-oriented evaluation (Balliet et al., 2014; Freeman and Ambady, 2011; Freeman et al., 2020). Decisions about whether to endorse or share symbolic content may also engage broader value-based and self-presentational processes (Scholz et al., 2020; Schlosser, 2020). However, behavioral measures alone do not indicate when the processing of these stimuli begins to diverge from the processing of ordinary objects, nor which stage of processing carries the most reliable distinction.

ERPs are well suited to this question because they can separate temporally distinct stages of processing with millisecond resolution (Amodio et al., 2014; Weber et al., 2015). Previous ERP studies have begun to examine self-referential and collective self-related processing elicited by national or identity-relevant cues. Fan et al. (2011) examined the temporal features of self-referential processing evoked by the national flag, and Shi et al. (2022) reported collective self-referential processing effects elicited by different national symbols. Related ERP work further suggests that individual and collective self-referential processing can differ in their temporal profiles (Liu et al., 2020), and that the importance of self-related social identities can modulate ERP responses over time (Xu et al., 2017). However, most of these studies compared different types of self-relevant or national-symbol stimuli with self-irrelevant stimuli, rather than directly comparing national-symbol icons with visually standardized neutral-object icons. Therefore, it remains unclear when national-symbol processing begins to diverge from neutral-object processing and which ERP time window carries the clearest category distinction.

On this basis, the present study focused on three predefined ERP time windows that map onto a plausible early-to-late temporal sequence, rather than assuming a fixed three-stage mechanism. The visual N1 was selected to capture early perceptual-attentional differentiation, although it can also vary with lower-level stimulus properties such as edge content, visual complexity, familiarity, and perceptual richness (Vogel and Luck, 2000; Hansen et al., 2011; Hu et al., 2022; Liu et al., 2022; Ofan et al., 2011; Pourtois et al., 2013). The 250–400 ms N400-range negativity was selected as the primary window because activity in this latency range is commonly associated with meaning-related processing and semantic or conceptual integration (Kutas and Hillyard, 1980; Kutas and Federmeier, 2011; West and Holcomb, 2002), and similar negativities have also been shown to vary with morally loaded meaning, norm-relevant information, partner-related knowledge, and other forms of socially meaningful content (Van Berkum et al., 2009; Mu et al., 2015; Salvador et al., 2020; Forgács et al., 2022; Liang et al., 2024; Rueschemeyer et al., 2015; Sinha et al., 2025). The LPP was included as a later, secondary window because it is broadly sensitive to sustained stimulus significance or evaluative engagement with motivationally relevant cues (Schupp et al., 2000, 2006; Hajcak et al., 2010; Hajcak and Foti, 2020; Fields, 2023; Zou et al., 2025), with related cue-reactivity and evaluative-processing work pointing in the same general direction (Moretta and Buodo, 2021; Paulmann et al., 2013; Wei et al., 2024).

Against this background, the present study used a standardized icon-based stimulus set to examine whether one culturally meaningful class of symbolic cues—national-symbol icons—would show temporally distinct neural responses relative to neutral-object icons. The use of line-drawing icons was intended to reduce, though not eliminate, low-level perceptual differences that often complicate comparisons based on naturalistic images. In addition to ERP measures, we assessed willingness to share the stimuli and subjective ratings of pleasantness, arousal, and patriotic feeling.

The present study addressed three related questions. First, do national-symbol icons elicit stronger behavioral responses than neutral-object icons, as reflected in sharing intention and subjective ratings of pleasantness, arousal, and patriotic feeling? Second, do national-symbol icons diverge from neutral-object icons across early, mid-latency, and late ERP time windows? Third, which ERP time window shows the clearest neural differentiation between the two stimulus categories? We hypothesized that national-symbol icons would elicit greater willingness to share and higher ratings of pleasantness, arousal, and patriotic feeling than neutral-object icons. At the neural level, we expected the clearest condition difference to emerge in the 250–400 ms window. We also examined whether an earlier N1 difference and a later LPP difference would be observed, with the N1 and LPP treated as secondary outcomes and interpreted cautiously.

## 2 Materials and methods

### 2.1 Participants

Thirty-four university students (18 men, 16 women; age range = 18–22 years, M = 19.82, SD = 0.75) participated in the study. All were right-handed, had normal or corrected-to-normal vision, and reported no history of neurological or psychiatric disorders. The study used a within-subject design, and all 34 participants were retained in the final analyses. To provide an explicit sample-size justification, we conducted a sensitivity power analysis using G*Power 3.1 (Faul et al., 2007, 2009) for the final sample. For a two-tailed paired-samples comparison with *α* = 0.05, power = 0.80, and *N* = 34, the minimum detectable effect size was approximately dz = 0.50. This sensitivity analysis indicates that the final sample provided adequate power to detect medium-sized within-subject condition effects, although smaller effects may have been underpowered. All participants were Chinese nationals who had been educated primarily in mainland China, which supported shared cultural familiarity with the national-symbol stimuli. Written informed consent was obtained before the experiment, and participants received financial compensation. The study was approved by the Ethics Committee of Science and Technology, Northeast Normal University (Approval No. 202502063), and all participants provided written informed consent before participation.

### 2.2 Materials

The experimental stimuli consisted of 60 black line-drawing icons divided into two categories: 30 national-symbol icons and 30 neutral-object icons. The national-symbol category included simplified icon-style depictions of nation-related symbols (e.g., the national flag, the national emblem, and the Great Wall), whereas the neutral-object category included icon-style depictions of everyday objects (e.g., cup, chair, clock, key, and umbrella). All stimuli were presented as black line drawings on a uniform gray background.

To reduce lower-level perceptual confounds, the two stimulus sets were constructed using the same visual language. All icons were standardized in canvas size, central position, line thickness, monochrome format, and the absence of shading, texture, and photographic detail. The stimuli were further designed to minimize scene-level variation by depicting single icon-like objects or symbols rather than naturalistic scenes or photographs. During stimulus construction, the icons were iteratively revised to improve comparability in occupied area, line density, and overall visual simplicity across categories. Although no formal independent norming study was conducted, these design procedures were intended to reduce, rather than eliminate, lower-level perceptual differences between categories. To provide quantitative information about low-level visual properties, objective image-level metrics were computed for each icon. Occupied area was calculated as the proportion of non-background pixels relative to the total image area, with the gray background estimated from the image corners. Edge density was calculated as the proportion of Canny edge pixels relative to the total image area. In addition, black-pixel proportion was calculated as the proportion of pixels substantially darker than the background. These metrics were compared between national-symbol and neutral-object icons using independent-samples t tests at the item level.

### 2.3 Procedure

The experiment employed a within-subject design and was conducted in a sound-attenuated and electrically shielded room. Participants were seated approximately 70 cm from the monitor. They first completed 8 practice trials to familiarize themselves with the task, followed by 120 formal trials divided into four blocks. A 2-min rest period was provided between blocks. The experiment lasted approximately 30 min. Practice trials used icon stimuli that did not appear in the formal experiment.

Each trial began with a fixation cross presented for 500 ms, followed by an icon presented for 2000 ms. All icons were presented centrally against the same gray background and subtended approximately the same visual angle across conditions. After a 1,000-ms blank screen, a prompt asking whether the participant would be willing to share the icon with others was presented. Participants responded by keypress, and the response screen remained visible until a response was made or until 5,000 ms had elapsed. This measure was intended to capture subjective sharing intention rather than actual sharing behavior.

**Figure 1:**
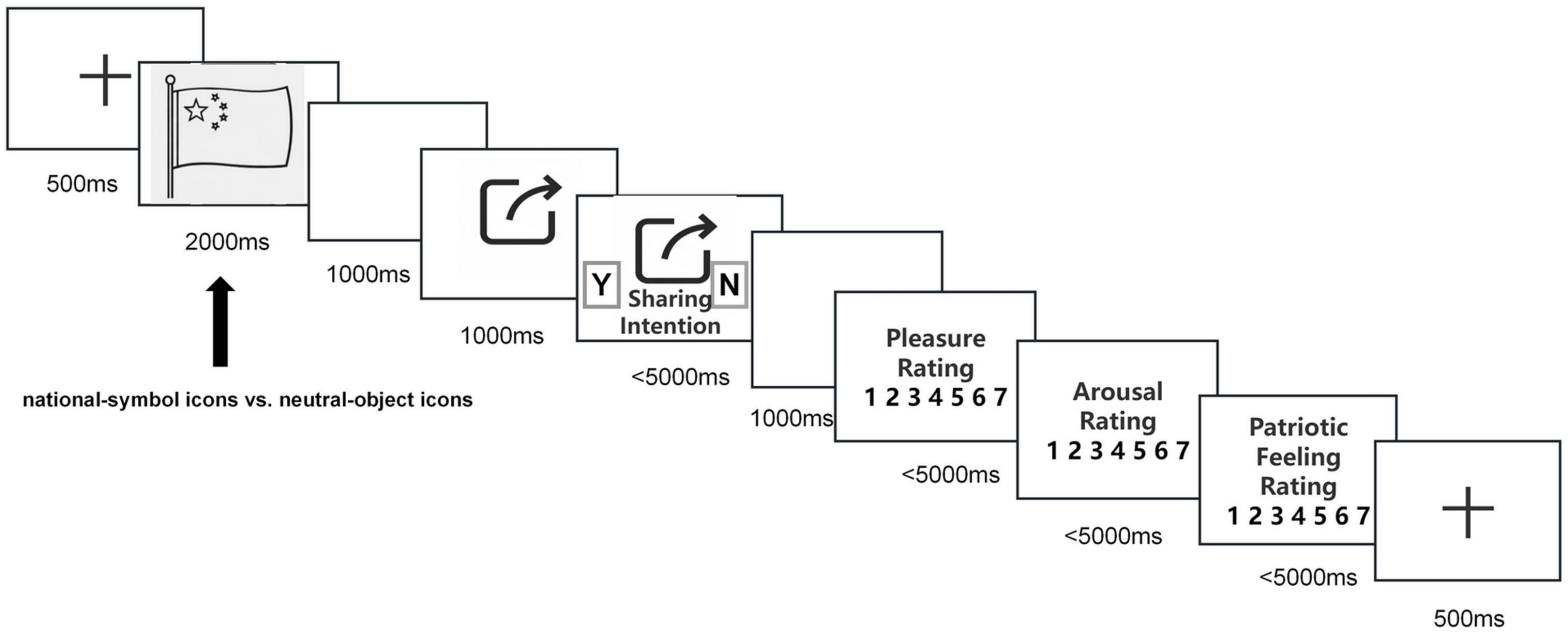
Schematic of the trial sequence. Each trial began with a fixation cross (500 ms), followed by icon presentation (2000 ms), a blank screen (1,000 ms), and a prompt asking whether the participant would share the icon. After this response, participants rated the icon on pleasantness, arousal, and patriotic feeling using 7-point scales.

After another blank screen, participants rated the icon on pleasantness, arousal, and patriotic feeling using 7-point scales. Pleasantness was rated from 1 (very unpleasant) to 7 (very pleasant), arousal from 1 (not arousing at all) to 7 (very arousing), and patriotic feeling from 1 (not at all) to 7 (very strongly). Each rating screen remained visible until a response was made or until 5,000 ms had elapsed. These ratings were used to assess the subjective significance of the stimuli alongside the binary sharing-intention response. Trial order was randomized separately for each participant (Figure 1).

### 2.4 EEG recording and preprocessing

EEG data were recorded using a 32-channel wireless Smarting Pro system (mBrainTrain, Belgrade, Serbia) with mbtStreamer (version 4.4.1) running on Windows. Signals were sampled at 250 Hz using a saline-based electrode cap arranged according to the international 10–20 system. Electrode impedances were kept below 20 kΩ throughout the experiment.

Stimulus presentation and behavioral responses were controlled using E-Prime 2.0. Event markers were sent from the presentation computer to the EEG recording stream at the onset of each icon presentation and were recorded in the event channel of the EEG data. The event-code scheme distinguished both stimulus category and stimulus subset. National-symbol icons were coded as 11, 12, and 13, whereas neutral-object icons were coded as 21, 22, and 23. These codes reflected different stimulus subsets within each category and were collapsed into national-symbol and neutral-object categories for ERP averaging and statistical analysis. First and second presentations of the same icon were not assigned separate event codes. Epochs were time-locked to these stimulus-onset markers. No additional hardware-based photodiode correction was applied; therefore, timing was based on software-generated event markers.

EEG data were processed in EEGLAB (version 14.1.1; Delorme and Makeig, 2004) under MATLAB R2016a (The MathWorks, Inc.). The data were re-referenced offline to the averaged mastoids and band-pass filtered from 0.1 to 30 Hz. Continuous EEG was segmented into epochs extending from −200 to 800 ms relative to these stimulus-onset markers. Independent component analysis (ICA) was performed using the extended ICA algorithm in EEGLAB with PCA reduction to 25 components. Artifact-related components were manually identified and removed by inspecting component scalp topographies, time courses, and activity patterns, with a focus on components reflecting eye blinks and horizontal eye movements. On average, 2.35 ICA components were removed per participant (SD = 0.85, range = 1–4). The 200-ms prestimulus interval was used for baseline correction. Trials were rejected if the voltage at any electrode exceeded ±100 μV relative to baseline. After artifact removal, ERPs were averaged separately for each stimulus category.

After artifact rejection, participants retained an average of 59.53 artifact-free trials (SD = 0.51) in the national-symbol icon condition and 59.65 artifact-free trials (SD = 0.49) in the neutral-object icon condition, out of 60 possible trials per condition. Thus, trial retention was very high and comparable across conditions. No participant was excluded because of insufficient usable trials.

### 2.5 Statistical analysis

Based on prior literature and the overall morphology of the grand-average waveforms, mean amplitudes were extracted for three predefined ERP time windows: N1 (100–150 ms), N400-range negativity (250–400 ms), and late positive potential (LPP; 550–750 ms). For the N1 and N400-range negativity, the frontal region of interest (ROI) included F3 and F4. For the LPP, the parietal ROI included P3 and P4. Mean amplitudes were averaged across the electrodes within each ROI for each condition and each participant.

Behavioral measures (sharing intention, pleasantness, arousal, and patriotic feeling) were compared between conditions using paired-samples t tests. For ERP data, repeated-measures analyses of variance (ANOVAs) were conducted separately for each time window, with Condition (national-symbol vs. neutral-object) as a within-subject factor and mean amplitude within the corresponding ROI as the dependent variable. Effect sizes are reported as partial eta squared (ηp^2^). All statistical analyses were conducted using SPSS 26.0, with the significance threshold set at *p* < 0.05. The primary ERP outcome was the condition difference in the 250–400 ms time window, whereas the N1 and LPP analyses were treated as secondary outcomes.

## 3 Results

### 3.1 Image-level control analyses

Objective image-level metrics were compared between the national-symbol and neutral-object icon sets. National-symbol icons showed a larger occupied area than neutral-object icons: national-symbol icons, M = 0.1289, SD = 0.0414; neutral-object icons, M = 0.0772, SD = 0.0241, *t*(58) = 5.910, *p* < 0.001. National-symbol icons also showed greater edge density than neutral-object icons: national-symbol icons, M = 0.0482, SD = 0.0168; neutral-object icons, M = 0.0260, SD = 0.0083, *t*(58) = 6.479, *p* < 0.001. In addition, national-symbol icons showed a higher black-pixel proportion than neutral-object icons: national-symbol icons, M = 0.1258, SD = 0.0404; neutral-object icons, M = 0.0754, SD = 0.0235, *t*(58) = 5.900, *p* < 0.001. These results indicate that, although the stimuli were standardized in format, residual low-level visual differences remained between the two icon sets.

### 3.2 Behavioral results

Behavioral responses differed reliably between the two stimulus categories. Participants reported a greater willingness to share national-symbol icons than neutral-object icons, *t*(33) = 12.092, *p* < 0.001, with mean sharing-intention scores of 0.64 (SD = 0.28) and 0.05 (SD = 0.10), respectively. National-symbol icons were also rated as more pleasant than neutral-object icons, *t*(33) = 10.003, *p* < 0.001, with mean ratings of 4.64 (SD = 0.86) and 2.42 (SD = 0.93), respectively. Similarly, arousal ratings were higher for national-symbol icons than for neutral-object icons, *t*(33) = 10.060, *p* < 0.001, with means of 4.58 (SD = 0.97) and 2.36 (SD = 0.94), respectively. Patriotic-feeling ratings were also higher for national-symbol icons than for neutral-object icons, *t*(33) = 10.422, *p* < 0.001, with means of 4.88 (SD = 0.90) and 2.46 (SD = 1.27), respectively. Together, these results indicate that national-symbol icons were associated with stronger subjective significance than neutral-object icons across all behavioral measures (Table 1).

**Table 1:** Behavioral responses to national-symbol and neutral-object icons.

Measure	National symbols, M (SD)	Neutral objects, M (SD)	*t*(33)	*p*
Sharing intention	0.64 (0.28)	0.05 (0.10)	12.092	<0.001
Pleasantness	4.64 (0.86)	2.42 (0.93)	10.003	<0.001
Arousal	4.58 (0.97)	2.36 (0.94)	10.060	<0.001
Patriotic feeling	4.88 (0.90)	2.46 (1.27)	10.422	<0.001

Sharing intention was coded as the proportion of trials on which participants indicated that they would share the icon. All *p* values reported in the table are two-tailed.

### 3.3 ERP results

**Figure 2:**
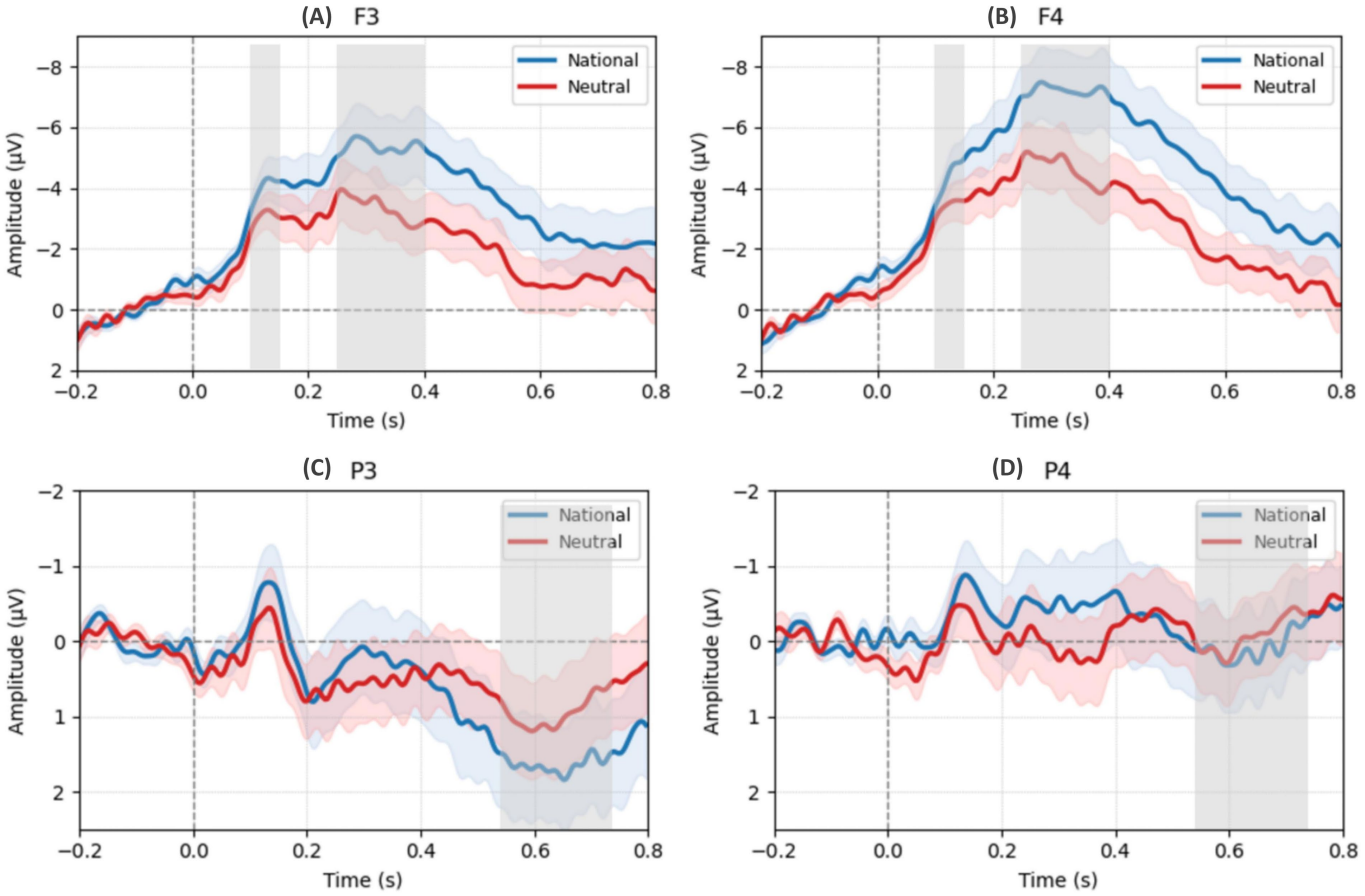
Grand-average ERP waveforms for national-symbol and neutral-object icons. **(A)** F3. **(B)** F4. **(C)** P3. **(D)** P4. Note: Grand-average ERP waveforms elicited by national-symbol and neutral-object icons at representative frontal and parietal electrode sites. Negative voltage is plotted upward. Shaded regions indicate the predefined time windows used for quantification: N1 (100–150 ms), N400-range negativity (250–400 ms), and LPP (550–750 ms). The displayed waveforms are shown for visualization purposes; statistical analyses were conducted on mean amplitudes extracted from the predefined frontal and parietal regions of interest.

**Figure 3:**
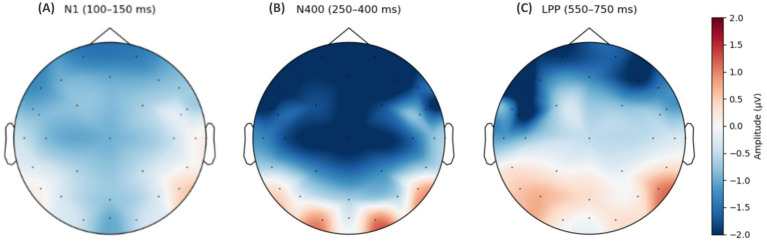
Topographic maps of condition differences across ERP time windows. **(A)** N1 (100–150 ms). **(B)** N400-range negativity (250–400 ms). **(C)** LPP (550–750 ms). Note: Difference waves were calculated as national-symbol minus neutral-object amplitudes. Each topographic map represents the mean voltage difference averaged within the corresponding predefined time window. Warmer and cooler colors indicate larger positive and negative differences, respectively.

Primary ERP outcome: N400-range negativity (250–400 ms). The clearest neural differentiation between stimulus categories emerged in the 250–400 ms time window (Figure 2). At the frontal ROI, national-symbol icons elicited a more negative mean amplitude than neutral-object icons, *F*(1, 33) = 23.22, *p* < 0.001, ηp^2^ = 0.413. Mean amplitude in this time window was more negative for national-symbol icons (M = −5.585, SD = 0.969) than for neutral-object icons (M = −3.561, SD = 0.819). As shown in the difference-wave topography (Figure 3), this effect was primarily distributed over frontal and central regions. This pattern indicates stronger mid-latency neural differentiation for national-symbol icons relative to neutral-object icons.

Secondary ERP outcome: N1 (100–150 ms). An earlier condition difference was also observed in the N1 time window. At the frontal ROI, national-symbol icons elicited a more negative N1 than neutral-object icons, *F*(1, 33) = 14.61, *p* = 0.001, ηp^2^ = 0.307. Mean N1 amplitude was −3.639 (SD = 0.522) for national-symbol icons and −2.765 (SD = 0.477) for neutral-object icons. This result suggests that category differentiation was already present at an early stage of processing.

Secondary ERP outcome: LPP (550–750 ms). In the later LPP time window, national-symbol icons showed numerically greater positivity than neutral-object icons at the parietal ROI. However, this difference did not reach statistical significance, *F*(1, 33) = 3.546, *p* = 0.069, ηp^2^ = 0.097. Mean LPP amplitude was 4.605 (SD = 1.134) for national-symbol icons and 4.139 (SD = 0.986) for neutral-object icons. Accordingly, the late positive potential should be interpreted cautiously and should not be treated as a primary finding of the present study.

## 4 Discussion

The present study examined the neural time course of one culturally meaningful class of socially meaningful symbolic cues by comparing national-symbol icons with neutral-object icons in a within-subject ERP task. Three findings were most notable. First, national-symbol icons were associated with greater willingness to share and higher ratings of pleasantness, arousal, and patriotic feeling. Second, national-symbol icons elicited both a more negative N1 and a larger N400-range negativity. Third, the clearest neural differentiation emerged in the 250–400 ms window, whereas the LPP difference did not reach statistical significance. Overall, the data suggest that national-symbol icons diverged from neutral-object icons in early and mid-latency ERP windows, with the strongest evidence arising at a mid-latency stage consistent with enhanced meaning-related processing.

The N1 difference indicates that category differentiation began relatively early. Although the two stimulus sets were presented in a standardized icon style, the image-level control analyses indicated that national-symbol icons had larger occupied area, greater edge density, and higher black-pixel proportion than neutral-object icons. Therefore, the N1 effect should be interpreted particularly cautiously and should not be treated as a selective index of symbolic meaning. Early visual ERP components remain sensitive to lower-level perceptual properties (Vogel and Luck, 2000; Hansen et al., 2011; Hu et al., 2022; Liu et al., 2022; Ofan et al., 2011; Pourtois et al., 2013), and the present design does not allow a clean separation of perceptual distinctiveness, visual complexity, category discrimination, and early prioritization of socially meaningful cues. The most defensible interpretation is therefore that national-symbol icons were differentiated earlier at perceptual-attentional stages, for reasons that may reflect both social significance and residual visual differences. This interpretation is also compatible with Frontiers evidence showing that the importance of self-related social identities can modulate the temporal features of ERP responses, suggesting that socially meaningful identity content may influence neural processing across multiple stages (Xu et al., 2017).

The strongest effect observed in the present study was the larger negativity elicited by national-symbol icons in the 250–400 ms time window. This pattern is most cautiously interpreted as reflecting enhanced meaning-related or conceptually elaborative processing for socially meaningful symbolic cues. In the ERP literature, neural activity in this latency range has been closely linked to semantic and conceptual integration (Kutas and Hillyard, 1980; Kutas and Federmeier, 2011; West and Holcomb, 2002), and recent work further suggests that similar negativities can be modulated by morally loaded meaning, norm-relevant information, partner-related knowledge, and other forms of socially meaningful content (Van Berkum et al., 2009; Mu et al., 2015; Salvador et al., 2020; Forgács et al., 2022; Liang et al., 2024; Rueschemeyer et al., 2015; Sinha et al., 2025). Compared with neutral-object icons, national-symbol icons are likely to evoke denser networks of culturally shared associations, including collective narratives, social values, historical knowledge, and identity-related meaning that extend beyond their immediate visual features. Accordingly, the present findings are consistent with the view that socially meaningful symbolic cues recruit stronger mid-latency processing related to the activation or integration of socially shared meaning. This interpretation is supported at the condition level by the behavioral findings: the same stimulus category that elicited the larger 250–400 ms negativity also received higher patriotic-feeling ratings and greater sharing intention. However, this condition-level convergence should not be interpreted as direct functional proof of meaning-related processing. The present study does not provide definitive participant-level brain–behavior validation linking the magnitude of the 250–400 ms ERP effect to individual differences in patriotic-feeling ratings; therefore, the interpretation of this effect remains cautious. The present design does not allow the 250–400 ms effect to be attributed uniquely to symbolic meaning, because symbolic significance was not fully dissociated from familiarity, affective salience, visual complexity, or broader category-level differences between national-symbol and neutral-object icons. Thus, the 250–400 ms effect is best understood as a mid-latency condition difference broadly compatible with meaning-related or conceptually elaborative processing, rather than as definitive evidence for a selective neural signature of symbolic meaning or national identification.

By contrast, the later LPP difference did not reach statistical significance and should therefore not be treated as a central finding of the present study. Although national-symbol icons showed numerically greater positivity than neutral-object icons, the current data do not provide strong evidence for a reliable late-stage difference in sustained evaluative processing. This cautious interpretation is important because the LPP is generally sensitive to stimulus significance in a broad sense, rather than uniquely indexing any single emotional state (Schupp et al., 2000, 2006; Hajcak et al., 2010; Hajcak and Foti, 2020; Fields, 2023; Zou et al., 2025). Related work in cue-reactivity and evaluative processing similarly suggests that late positive activity can reflect continued engagement with motivationally relevant stimuli (Moretta and Buodo, 2021; Paulmann et al., 2013; Wei et al., 2024). In the present data, however, the LPP pattern remains secondary to the stronger effect observed in the 250–400 ms time range and should not be treated as a principal result.

More broadly, these findings extend prior self-report and behavioral work by providing temporally resolved evidence about when socially meaningful symbolic cues begin to diverge from neutral objects. The results should not be taken as identifying a definitive neural mechanism of national identity. Rather, they suggest that one class of culturally shared symbolic cues is processed differently from neutral objects, with the most reliable divergence appearing in a time range often linked to meaning-related or conceptually elaborative processing.

From a broader human-neuroscience perspective, this temporal profile is informative because it suggests that the processing advantage of socially meaningful symbolic cues is not confined to *post hoc* evaluation or explicit judgment. Instead, the ERP pattern implies an ordered sequence in which early perceptual-attentional differentiation is followed by a more robust mid-latency effect compatible with the activation or integration of socially shared meaning. This sequence helps locate the level at which such cues begin to influence processing and provides a useful framework for future studies aimed at disentangling perceptual, affective, mnemonic, and conceptual contributions to responses toward culturally meaningful symbols.

Several limitations should be noted. First, the symbolic stimuli in this study were limited to Chinese national-symbol icons, and the sample consisted exclusively of Chinese university students. Accordingly, the present findings should not be interpreted as applying only to nationalism-related processing, but rather as an initial demonstration using one culturally meaningful class of socially shared symbolic cues. Future research should examine whether similar temporal patterns emerge for other identity-relevant or socially meaningful symbolic stimuli across different cultural contexts and participant groups. Second, symbolic meaning was not fully isolated from potentially confounding factors, including familiarity, affective salience, visual complexity, and broader category-level differences between national-symbol and neutral-object icons. The image-level control analyses showed that national-symbol icons had larger occupied area, greater edge density, and higher black-pixel proportion than neutral-object icons. These residual low-level visual differences are particularly relevant for interpreting the early N1 effect and may also have contributed to later condition differences. Therefore, although the present design allowed us to detect reliable condition differences, tighter stimulus matching and independent norming will be necessary to determine more precisely which stimulus properties drove the observed ERP effects. Third, the functional interpretation of the ERP components should be made cautiously. In particular, the effect observed in the 250–400 ms time window is consistent with enhanced meaning-related or conceptual processing, but ERP measures alone do not allow definitive conclusions about the exact cognitive mechanisms involved. Converging evidence from more tightly controlled behavioral paradigms, additional neurophysiological measures, or cross-cultural designs would help clarify the psychological processes underlying these effects. Fourth, although national-symbol icons received higher patriotic-feeling ratings than neutral-object icons at the condition level, the present study does not provide definitive participant-level brain–behavior validation of the 250–400 ms effect. Future research should directly test whether individual differences in mid-latency ERP differentiation are associated with trial-level or participant-level variation in patriotic feeling, symbolic meaning ratings, or other behavioral indices of subjective significance. Fifth, the subjective ratings were always collected in a fixed order: pleasantness, arousal, and patriotic feeling. This fixed order may have introduced carryover effects among the rating measures, especially because pleasantness and arousal judgments preceded patriotic-feeling ratings. Importantly, however, the ERP responses were time-locked to icon onset and were recorded before the rating screens appeared; therefore, the rating order could not have influenced the ERP effects, although it may have affected the subjective rating pattern.

## 5 Conclusion

In summary, national-symbol icons were differentiated from neutral-object icons at both behavioral and neural levels, with the clearest neural effect occurring in the predefined 250–400 ms time window. The pattern is broadly consistent with enhanced mid-latency processing of socially shared meaning in one culturally meaningful class of symbolic cues. At the same time, this interpretation remains tentative because the present study provides condition-level behavioral convergence rather than definitive participant-level brain–behavior validation. More tightly controlled and cross-cultural studies will be needed to clarify which stimulus properties drive these effects and how generalizable the pattern is.

## Data Availability

The datasets supporting the conclusions of this article will be made available by the author upon reasonable request, subject to ethical restrictions related to participant confidentiality.
